# Elder abuse and the association with ear or hearing diseases in advanced age: a cross-sectional study

**DOI:** 10.1186/s12877-026-07222-2

**Published:** 2026-02-24

**Authors:** Tino Prell, Antonia Wagner, Aline Schönenberg, Konstantin G. Heimrich

**Affiliations:** 1 Department of Geriatrics, Halle University Hospital, Halle (Saale), Germany; 2 Department of Geriatrics, Jena University Hospital, Am Klinikum 1, Jena, 07747 Germany

**Keywords:** Aged abuse, Hearing impairment, Oldest-old, Risk factors, Quality of life

## Abstract

**Background:**

Elder abuse (EA) is a serious health issue in older adults. The risk of EA may be increased by ear or hearing diseases (EHD), which are prevalent in this population and can lead to dependence on caregivers. The objective of the study was to determine the association between EHD and EA in older adults.

**Methods:**

Data were derived from 2,817 individuals aged 80 years or older from the Old Age in Germany (D80+) study. EA was measured using the Elder Abuse and Emotional Consequences Scale (EACS) and its subscales: intimidation, shaming and blaming, paternalism, neglect, financial exploitation, and physical behavior. Elastic net regressions and multivariate analyses of covariance (MANCOVA) were performed to examine the link between EHD and EA subscales.

**Results:**

Individuals had a mean age of 84.4 ± 3.9 years. Of these, 52.4% were male and 47.6% female. The prevalence of EA was higher in individuals with EHD (84.2%) compared to those without (79.4%, *p* < .001). In addition to depressive symptoms (coefficient = 0.101, *p* < .001), EHD was also significantly associated with EA (coefficient = 0.052, *p* = .003). MANCOVA results showed a multivariate effect of EHD on EA subscales (Wilks’ λ = 0.987, F(6, 2653) = 5.696, *p* < .001, partial η² = 0.013). The strongest effects were observed on the intimidation (F = 22.364, *p* < .001, η² = 0.008) and neglect subscales (F = 21.151, *p* < .001, η² = 0.008).

**Conclusions:**

The study identified an association between EHD and EA. This emphasizes the importance of addressing hearing impairments in EA prevention strategies.

**Supplementary Information:**

The online version contains supplementary material available at 10.1186/s12877-026-07222-2.

## Introduction

Elder abuse (EA) is a serious issue affecting the health and well-being of older adults. As defined by the World Health Organization (WHO), EA is a single or repeated act or lack of appropriate action, occurring within a relationship where there is an expectation of trust, that causes harm or distress to an older person [1]. It includes physical, emotional, sexual, and financial abuse, as well as neglect. Globally, 10–15% of community-dwelling older adults experience EA, with psychological abuse, financial abuse, and neglect being most common [2]. In institutional settings, psychological abuse is most frequently reported (33.4%), followed by physical, financial, neglect, and sexual abuse [3]. EA can lead to serious outcomes, including poor health, hospitalization, and psychological distress [4, 5]. Understanding its prevalence and causes is crucial for prevention, especially as the population ages.

Age-related hearing loss is common in older adults and can severely affect daily life and social interactions [6]. Beyond communication difficulties, hearing impairment is linked to social isolation, cognitive decline, and mental health issues like depression and anxiety [7, 8]. It can hinder participation in social activities, increase feelings of loneliness, and impair daily functioning [9, 10]. Misunderstandings and frustration may also escalate the risk of abuse, leaving individuals vulnerable to neglect and exploitation.

The intersection of EA and hearing impairment warrants further research. Older individuals who are deaf or hard of hearing face unique challenges in communicating their needs, increasing their vulnerability to abuse [11]. Their reliance on caregivers can heighten dependency, risking exploitation or neglect [12]. Social isolation due to hearing loss also reduces access to support systems, amplifying abuse risk [13]. However, the precise link between hearing impairment in advanced age and different domains of EA is underexplored. Accordingly, the primary objective of our study is to examine the association between EHD and the different subscales of the EACS. It is important to investigate this association to gain insight into the distinctive challenges faced by this population so that targeted interventions can be developed to mitigate risks and improve outcomes for hearing impaired older people.

As hearing impairment rises in aging populations, studying its link to EA is crucial. This research will deepen our understanding of the vulnerabilities of older adults with hearing impairments and help develop strategies to prevent abuse and enhance the quality of life of this population.

## Methods

### Sample

For this study, data from the “Old Age in Germany (D80+)” survey, a nationally representative sample of registered individuals aged 80 years and older (born before 1 March 1940; with no upper age limit) in Germany were used. The cross-sectional data were derived from a written questionnaire (module 1) and a telephone survey (module 2). Both community-dwelling and institutionalized participants, or their proxies, were interviewed. This approach also included people who were unable to provide their own information due to illness or disability to avoid positive bias. The study was conducted between November 2020 and October 2021 by the University of Cologne in collaboration with CERES (Cologne Center for Ethics, Rights, Economics, and Social Sciences of Health) and the German Centre of Gerontology (DZA). The study was funded by the Federal Ministry for Family Affairs, Senior Citizens, Women and Youth (BMFSFJ). Study details are available at www.dza.de/forschung/fdz/d80/doi/d80-2022-m001. The multi-stage sampling process involved randomly selected municipalities and resident registration data, with samples stratified by gender and age. For the purpose of the study, a total of 40,209 people were contacted. Of these, 10,360 returned the questionnaire and were included in module 1 of the study. In a second step, a telephone survey was conducted with 3,233 participants to gain further information (module 2, interview), including information regarding elder abuse. The majority of the data was collected using self-report (model 1). However, it was possible to nominate a relative, friend, legal representative, or caregiver for the telephone survey (model 2), for which 322 proxies were interviewed. More methodological details regarding the representativeness of the sample, data weighting, and the scientific use file are available in corresponding method papers [14, 15].

### Dependent variable

The variable of interest was EA as measured by the previously developed Elder Abuse and Emotional Consequences Scale (EACS), which is a validated instrument for the multidimensional assessment of EA in advanced age [4, 16]. The scale measures experiences of EA and their emotional impact. It comprises 15 items and captures the frequency and type of these experiences within the past 12 months from the perspective of older adults using a 5-point Likert-style response format (0 = never; 1 = seldom; 2 = sometimes; 3 = often; 4 = very often).

For descriptive statistics, a dichotomous outcome variable for EA was constructed. In line with previous literature, elder abuse was defined as present, if the interviewed person rated at least one of the 15 items as present (seldom, sometimes, often, or very often) [16]. In addition, we calculated the sum of the 15 EACS items as an overall measure of EA (EA sum score) for regression analysis. To gain a more nuanced view of elder abuse experiences, we utilized six subscales that target specific domains of abuse. Therefore, each scale value was calculated by adding the assigned items with a weighting corresponding to the factor loading as given in the D80 + Manual or previous literature [16]. In general, higher scores indicate greater frequency and severity in that domain. The six subscales of the EACS are [16]:

- Intimidation: Items addressing instances of threats or verbal aggression.
- Shaming and blaming: Focuses on scenarios where individuals feel unjustly accused or criticized.
- Paternalism: Evaluates controlling or infantilizing behaviors directed at the individual.
- Neglect: Captures experiences of being ignored or having unmet basic needs.
- Financial exploitation: Relates to mismanagement or misuse of financial resources by others.
- Physical behavior: Encompasses unwanted or harmful physical contact.

### Independent variable and covariates

Our main independent variable of interest was the presence [1] or absence (0) of ear or hearing disease (EHD), as assessed by a single-item question provided to participants, and its influence on EA. In addition, we included the following 12 covariates:

- Age, gender (assessed by questionnaire).
- Social network: captures the number of people in a respondent’s social network (self-reported number), reflecting the size of their immediate social support system (assessed by questionnaire). Larger values indicate a broader network of potential social support [17].
- Level of education (ISCED): classifies educational attainment into three categories based on the International Standard Classification of Education (ISCED) (assessed by interview): 1 = Low (ISCED 0–2, respondents without completed vocational qualifications and without A-levels [Abitur]), 2 = Medium (ISCED 3–4, respondents with vocational qualifications or respondents with A-levels [Abitur]), 3 = High (ISCED 5–6, respondents with university degrees or master craftsmen).
- Full inpatient care: indicates whether participants reside in institutionalized full-care settings (0 = No, 1 = Yes) (assessed by questionnaire and validated by interview).
- Type of housing: differentiates participants living in private residences or outpatient assisted house and flat-sharing communities (0) from those in retirement homes, nursing homes, residential care facilities, or residential care groups [1] (assessed by interview).
- Number of medications: represents the count of prescribed medications per day regularly taken by participants (assessed by questionnaire), which can serve as an indicator of overall health and multimorbidity [18].
- Functional health (ADL): assesses basic activities of daily living (ADL) (e.g., eating, dressing), derived from seven items using a 3-item Likert scale (0, only with help possible; 1, with little help; 2, no help) (assessed by interview). Higher scores (ranging from 0 to 14) denote better functioning [19].
- Functional health (IADL): evaluates instrumental activities of daily living (IADL) (e.g., managing finances, medication use) from seven items recorded using a 3-item Likert scale (0, only with help possible; 1, with little help; 2, no help) (assessed by interview). Higher scores (ranging from 0 to 14) reflect better performance in these activities [20].
- Cognition (semantic fluency): measures participants’ ability to generate words within a category over a set time (within one minute, the participant has to name as many things as possible that can be bought at the supermarket) (assessed by interview). Scores are raw counts of words produced between 0 and 60 [21].
- Cognition (delayed recall): assesses delayed memory retention after reading a list of 10 words to the participant (assessed by interview); scored between 0 and 10. Higher scores signify better recall ability.
- Depression (DIA-S4): assesses four depressive symptoms regarding emotional and physical states (0 = No, 1 = Yes) (assessed by interview). The sum scores range from 0 to 4 with higher scores suggesting greater depressive symptoms; score between 2 and 4 indicated depression [22].

For more information regarding the provided information on the used covariates, we refer to the methodological details and the scientific use file in the corresponding method papers of the “D80+” study [14, 15].

### Statistical analyses

The statistical analysis was conducted using IBM SPSS Statistics (Version 27) and R (4.1.1). Descriptive statistics were used to initially characterize the participants. We performed independent group comparisons for two groups using the U-test and chi-square tests. Effect sizes of the group differences were determined using the rank biserial correlation for the U-test and Cramer’s V for the chi-square test.

Elastic net regression was used to assess the relationship between EHD and EA, with EA as a binary outcome (logistic regression) or a sum score (linear regression). The elastic net regression combines the strengths of the Lasso (variable selection) and Ridge (stability) regularization method to improve generalization and prevent overfitting. Besides EHD as independent variable, the following 12 covariates were included: Age, gender, level of education, full inpatient care, type of housing, social network, number of medications, functional health (ADL, IADL), depressive symptoms, and cognition (semantic fluency, delayed recall). The *glmnet* package (4.1–7.1) implemented this regression combining Lasso (L1) and Ridge (L2) penalties. Missing values were handled by listwise deletion, and cross-validation determined the optimal lambda. A final elastic net model was fitted using this lambda value to identify predictors with non-zero coefficients (L1), which were further refined with an Ordinary Least Squares (OLS) regression (L2). This two-step approach enabled a robust exploratory analysis of the selected predictors.

A multivariate analysis of covariance (MANCOVA) was performed to investigate the impact of EHD on the six EA subscales (intimidation, shaming and blaming, paternalism, neglect, financial exploitation, and physical behavior). Wilks’ lambda and partial η^2^ values quantified the effects [23].

All tests were two-tailed, with statistical significance set at *p* <.05.

## Results

### Characteristics of the sample

For this analysis, all participants of the “Old Age in Germany (D80+)” survey with complete EACS were included (*N* = 2,817), corresponding to 87% of the interviewed participants (*N* = 3,233). Description of the cohort is given in Table 1. The cohort is characterized by a broad variability in the number of social contacts and varying levels of independence, due to the inclusion of both community-dwelling and institutionalized participants. The mean age was 85.4 (SD = 3.9, range 80 to 100) years. The gender distribution is nearly balanced, with a slightly higher proportion of males (*N* = 1,475, 52.4%). Educational attainment is diverse, with the majority having medium-level qualifications. Most participants reside in private living arrangements, with a small proportion in institutional settings or full residential care. Depressive symptoms are prevalent in about one-third of the cohort. In the studied cohort, EHD was prevalent in 981 individuals (34.8%); 1,744 individuals (61.9%) reported no EHD (*N* = 92 cases with missing values). People with EA were younger, more depressed and reported more often ear or hearing diseases (Table 1).

**Table 1:** Cohort’s characteristics and group comparison

Variable (metric, ordinal)	Group	*N*	Mean	SD	Median	IQR		*p*	*r*
						Q1	Q3		
Age	total	2,817	85.41	3.93	84.00	82.00	87.50		
	EA absent	531	86.02	4.21	85.00	82.00	89.00	< 0.001	0.100
	EA present	2,286	85.26	3.85	84.00	82.00	87.00		
	Missing	0							
Social network	total	2,793	8.77	7.15	6.00	4.00	12.00		
	EA absent	524	8.36	6.94	6.00	3.00	12.00	0.072	/
	EA present	2,269	8.86	7.20	6.00	4.00	12.00		
	Missing	24							
Number of medications	total	2,600	5.09	3.36	4.00	3.00	7.00		
	EA absent	489	4.92	3.16	4.00	3.00	6.00	0.248	/
	EA present	2,111	5.13	3.41	5.00	3.00	7.00		
	Missing	217							
Functional health (ADL)	total	2,816	1.85	0.29	2.00	1.86	2.00		
	EA absent	531	1.85	0.28	2.00	1.86	2.00	0.631	/
	EA present	2,285	1.85	0.29	2.00	1.86	2.00		
	Missing	1							
Functional health (IADL)	total	2,793	1.69	0.43	1.86	1.57	2.00		
	EA absent	525	1.69	0.44	1.86	1.57	2.00	0.332	/
	EA present	2,268	1.69	0.43	1.86	1.57	2.00		
	Missing	24							
Cognition (semantic fluency)	total	2,675	18.62	6.74	18.00	14.00	23.00		
	EA absent	500	18.41	6.80	18.00	14.00	23.00	0.419	/
	EA present	2,175	18.67	6.72	18.00	14.00	23.00		
	Missing	142							
Cognition (delayed recall)	total	2,571	4.57	2.68	5.00	3.00	6.00		
	EA absent	467	4.57	2.69	5.00	3.00	6.00	0.974	/
	EA present	2,104	4.56	2.68	5.00	3.00	6.00		
	Missing	246							
Variable (nominal)	Group	total		EA absent		EA present		p	r
		N	%	N	%	N	%		
Gender	total			531	18.8	2,286	81.2	0.246	/
	male	1,475	52.4	266	18.0	1,209	82.0		
	female	1,342	47.6	265	19.7	1,077	80.3		
	Missing	0	0.0						
Level of education	total			518	18.8	2,242	81.2	0.189	/
	low	321	11.4	70	21.8	251	78.2		
	medium	1,382	49.1	264	19.1	1,118	80.9		
	high	1,057	37.5	184	17.4	873	82.6		
	Missing	57	2.0						
Full inpatient care	total			529	18.9	2,274	81.1	0.933	/
	no	2,683	95.2	506	18.9	2,177	81.1		
	yes	120	4.3	23	19.2	97	80.8		
	Missing	14	0.5						
Type of housing	total			531	18.8	2,286	81.2	0.913	/
	private	2,776	98.5	523	18.8	2,253	81.2		
	nursing	41	1.5	8	19.5	33	80.5		
	Missing	0	0.0						
Depression (DIA-S4)	total			483	18.9	2,077	81.1	< 0.001	0.119
	no	1,812	64.3	396	21.9	1,416	78.1		
	yes	748	26.6	87	11.6	661	88.4		
	Missing	257	9.1						
Ear or hearing diseases	total			515	18.9	2,210	81.1	0.002	0.059
	no	1,744	61.9	360	20.6	1,384	79.4		
	yes	981	34.8	155	15.8	826	84.2		
	Missing	92	3.3						

Values are presented as mean (M), standard deviation (SD), and Median and interquartile ranges (IQR); categorical parameters are presented as numbers (N) and percentages (%). For group comparisons, U-tests and chi-square tests were performed. Effect sizes were given as rank biserial correlation and Cramer’s V

*ADL* basic activities of daily living, *DIA-S4* Depression In old Age Scale with 4 items, *EHD* ear or hearing diseases, *IADL* instrumental activities of daily living

*P*-values refer to the comparison of people with and without elder abuse (EA)

### Prevalence and patterns of EA

When EA was defined dichotomously [16], EA was more common in people with EHD (*N* = 826, 84.2%) than in people without EHD (*N* = 1,384, 79.4%) (*p* =.002, Cramer´s V = 0.06).

All EA subscales were significantly higher in people with EHD compared to people without EHD (Supplementary Table 1).

**Fig. 1:**
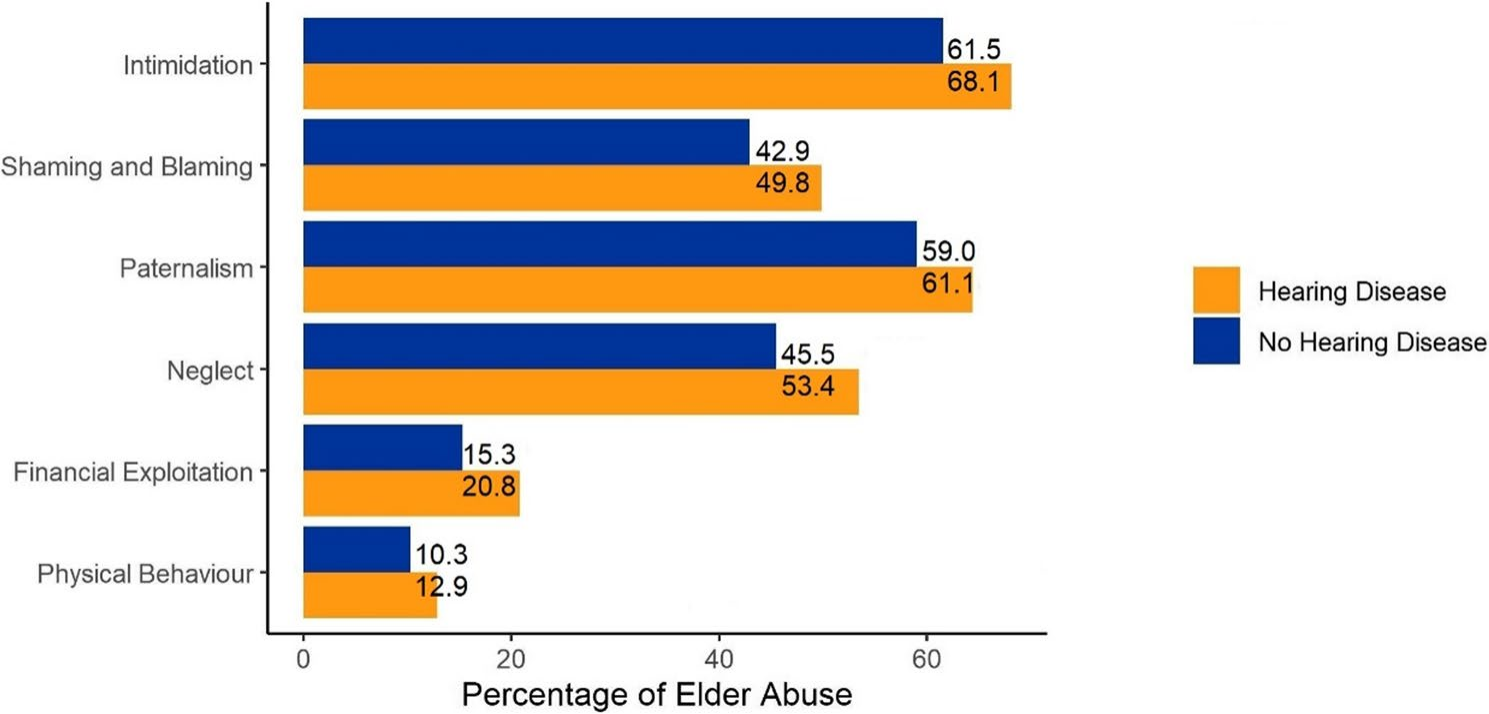
Frequency of elder abuse patterns in people with and without ear or hearing diseases

The most prevalent form of EA in the current analysis was intimidation, with a prevalence of 61.5% among individuals without EHD and 68.1% among those with EHD (*p* <.001) (Fig. 1, detailed in Supplementary Table 2). Paternalism was the second most frequent type, observed in 59.0% of individuals without EHD and 64.4% of those with EHD (*p* =.006). Neglect followed closely, affecting 45.5% of individuals without EHD and 53.4% of those with EHD (*p* <.001). Shaming and blaming was also prevalent, with rates of 42.9% and 49.8% in these respective groups (*p* <.001). Financial exploitation was less frequent, reported by 15.3% of individuals without EHD and 20.8% of those with EHD (*p* <.001). The least common form of EA was physical behavior, occurring in 10.3% of individuals without EHD and 12.9% of those with EHD (*p* =.047) (Fig. 1, detailed in Supplementary Table 2). Analyses on item level illustrate that the strongest differences are observed for items like “Become louder” (+ 7.5%), “Did not give time” (+ 7.2%), and “Blamed for an event” (+ 6.9%) (Supplementary Table 3; Supplementary Fig. 1).

Prevalence of elder abuse (%) in people with and without ear or hearing diseases regarding the six subscales of the Elder Abuse and Emotional Consequences Scale (EACS).

### EHD is associated with EA

A logistic regression elastic net model was employed to ascertain predictors of EA among EHD and a comprehensive set of 12 covariates, encompassing demographic, health-related, and functional measures. The results indicated a significant positive association between EA and EHD (coefficient = 0.052, SE = 0.018, *p* =.003), indicating that individuals with EHD are at an elevated risk of experiencing EA. In contrast, age exhibited a significant negative association with EA (coefficient = −0.008, SE = 0.002, *p* <.001). This suggests that as age increases, the probability of EA declines, although the effect size is relatively modest. Moreover, depression was identified as the most significant predictor of EA in the model (coefficient = 0.101, SE = 0.018, *p* <.001). However, the model exhibited a relatively low overall explanatory power (corrected R^2^ = 0.022, *F*(3, 2061) = 16.78, *p* <.001). The aforementioned findings were corroborated through a linear regression analysis, wherein the effects of potential confounding variables were controlled. This analysis revealed a statistically significant association between EHD and the EA sum score (corrected R² = 0.10, *F*(13, 2051) = 18.58, *p* <.001; see Supplementary Table 4).

Finally, a MANCOVA was used to evaluate the association between hearing disorders and the six subscales of the EACS, encompassing intimidation, shaming and blaming, paternalism, neglect, financial exploitation, and physical behavior (see Supplementary Table 5). A significant multivariate effect of ear or hearing diseases was observed (Wilks’ lambda = 0.987, *F*(6, 2653) = 5.696, *p* <.001, partial η^2^ = 0.013), indicating a small but statistically significant overall effect on the subscales. Among the subscales, EHD had the most pronounced impact on intimidation (*F*(1, 2658) = 22.364, *p* <.001, partial η^2^ = 0.008) and neglect (*F*(1, 2658) = 21.151, *p* <.001, partial η^2^ = 0.008), both showing the highest effect sizes. This indicates that individuals with EHD are more likely to experience intimidation and neglect than those without such impairments. Furthermore, other subscales, including shaming and blaming, paternalism, financial exploitation, and physical behavior, were also found to be significantly affected (*p* <.01), albeit with smaller effect sizes, with partial η^2^ values ranging from 0.003 to 0.006.

## Discussion

This study examined the association between EHD and EA, providing valuable insights into this hitherto underexplored topic. The findings indicate that while EHD was associated with an increased risk of EA, additional factors such as age and depressive symptoms were also identified as important variables associated with EA. These findings highlight the importance of understanding the complex nature of elder abuse, particularly among vulnerable persons with hearing impairments. However, the results also show that EA is common and can occur regardless of gender, social network size, level of education, multimorbidity, or physical and cognitive limitations. EA can affect both community-dwelling people and those in institutionalized care. Therefore, elder abuse is a widespread issue that generally requires special attention.

The prevalence of EA can vary depending on the definition and context used in the relevant studies. Notwithstanding, the prevalence of EA in our study was higher than that reported in previous reviews [2, 3]. Accordingly, it is also important to consider the underlying survey instrument. The EACS is a relatively new assessment tool based on the WHO definition of elder abuse. It was developed and evaluated using a German cohort of individuals with a minimum age of 80 years [4, 16]. A previous study in North Rhine-Westphalia (NRW) revealed that approximately half of the sample (54.1%) had experienced a form of EA at least once during the previous 12 months [4]. The current analysis revealed a prevalence of EA between 79 and 84%. Thereby, the most prevalent form of EA in the current analysis was intimidation, followed by paternalism and neglect. Similarly, Brijoux et al. observed that psychological abuse and neglect were more prevalent than physical or financial abuse [4]. In addition to the assessment tool, the studied cohort is also decisive when comparing prevalence of EA or its subdomains. In this regard, the prevalence of psychological abuse in people with dementia can already be estimated at up to 62% [24]. Therefore, it is recommended that the EACS is examined in further subpopulations to investigate the underlying differences in EA prevalence and to identify specific risk groups.

Brijoux et al. identified higher rates of multimorbidity, smaller social network size, and increased aggression directed towards the victim as significant predictors of EA [4, 16]. In alignment with the findings of the aforementioned study, no significant correlation was identified between EA and factors such as cognitive status, education, and gender. In accordance with the findings of our study, other studies have also identified a correlation between depressive symptoms and EA [2, 4]. In contrast with the findings of Brijoux et al., our results indicated that age may act as a protective factor, suggesting that younger individuals within our oldest-old cohort may be more susceptible to EA. A reason for this cannot be specified. However, one possible explanation is that younger older adults often have greater independence and social engagement, which may increases their exposure to potentially abusive situations, especially when they become partially dependent on care. This can result in heightened psychosocial stress, which is linked to EA, particularly when limitations emerge [25]. Unlike the oldest old, who more commonly reside in institutional care settings with structured supervisions, this group frequently relies on family members or non-institutional caregivers, which may heighten the risk of abuse. Additionally, intergenerational or familial conflicts related to caregiving responsibilities may further exacerbate abuse risks [26, 27]. Although the oldest old are usually more frail and cognitively impaired, which limits their ability to report abuse, they often receive more consistent professional supervision, which may mitigate abuse. These dynamics underscore the necessity of targeted screening and protective interventions tailored to the distinct vulnerabilities of different elderly age subgroups. However, the deviating association between age and EA cannot be assessed conclusively and requires further investigation.

The weak but significant association between EHD and EA observed in our study is consistent with the findings of Brijoux et al., who identified multimorbidity as a risk factor for EA [4]. Nevertheless, the modest effect size observed in both studies highlights the multifactorial nature of EA, whereby sensory impairments, such as hearing difficulties, may represent one component of a broader vulnerability and multimorbidity profile. Our findings reinforce the necessity for further investigation into the specific mechanisms through which sensory impairments contribute to EA risk, as well as the development of targeted interventions to mitigate these risks in older adults.

The weak but significant direct and indirect association between EHD and EA identified in our study has significant implications for further research. It underscores the potential role of EHD as a contributing factor to vulnerability in older adults. The communication barriers associated with EHD may hinder individuals from reporting abuse or seeking assistance, while simultaneously increasing their reliance on caregivers. Furthermore, behaviors related to communication, such as difficulties in understanding or expressing needs, should be considered to gain a more comprehensive understanding of the potential mediators of this effect. Furthermore, the general quality of social relationships is of great consequence and requires further investigation to ascertain the variables through which this association operates. These findings suggest that hearing problems should be considered as a risk factor in EA prevention strategies, especially in health and social service settings.

This study is not without limitations. First, as this is a cross-sectional study, it is not possible to establish any causal relationships beyond the associations described. In particular, it is not possible to determine whether EHD exerts a negative influence on EA or whether there is reverse causality. Accordingly, further longitudinal studies are necessary to investigate causalities between EHD and EA, and to determine whether EA mediates the relationship between hearing problems and adverse health outcomes over time. Second, selection bias cannot be ruled out. Approximately one fourth of those contacted agreed to participate in the study. In this regard, no conclusions can be drawn about different biopsychosocial factors between participants and individuals who decided not to participate. However, a high percentage of 87% of the participants interviewed answered the EACS questions. These participants were described in detail to enable comparability in future studies. Third, due to the minimum age of 80 years, the results are not generalizable. Accordingly, the results cannot be transferred directly to younger older people (< 80 years of age). Fourth, most of the data were collected through self-reports, with additional proxy reports used when applicable. Although proxies are often indispensable when participants are unable to provide reliable information themselves, differences in perception and reporting accuracy may have affected our findings. In particular, proxy respondents tend to underestimate subjective experiences [28–30]. This can lead to differential misclassification. In contrast, self-reports may be affected by the respondents’ motivation and recall bias [31], but they generally reflect an individual’s internal experiences more accurately. Consequently, using a combination of proxies and self-reports could have introduced heterogeneity into the dataset, affecting the magnitude and direction of the observed associations. Finally, the severity and degree of EHD, as well as the functional impairment associated with it, were not specified. Further research should investigate whether the severity of hearing loss is associated with specific types of abuse or moderates other outcomes, such as depression and reduced quality of life. Closing these gaps will improve our understanding of the complex relationship between hearing impairments and EA.

## Conclusion

The findings of this study provide evidence of a weak yet significant association between EHD and EA. Elucidating this association lays the groundwork for future research investigating how improving hearing problems through screening and individualized treatment strategies can be incorporated into EA prevention strategies and how this can impact public health and social care.

## Supplementary Information

Supplementary Material 1.

## Data Availability

The datasets used and/or analysed during the current study are available from the DZA on reasonable request (http://www.dza.de).

## Abbreviations

ADL: Activities of daily living
BMFSFJ: Federal Ministry for Family Affairs, Senior Citizens, Women and Youth
CERES: Cologne Center for Ethics, Rights, Economics, and Social Sciences of Health
DZA: German Centre of Gerontology
EA: Elder abuse
EACS: Elder Abuse and Emotional Consequences Scale
EHD: Ear or hearing diseases
IADL: Instrumental activities of daily living
ISCED: Level of education
MANCOVA: Multivariate analyses of covariance
OLS: Ordinary Least Squares
WHO: World Health Organization

## References

[1] WHO. A global response to elder abuse and neglect: building primary health care capacity to deal with the problem world-wide: main report. 2008 [zitiert 26. Juni 2025]. Verfügbar unter: https://www.who.int/publications/i/item/9789241563581.

[2] Yon Y, Mikton CR, Gassoumis ZD, Wilber KH. Elder abuse prevalence in community settings: a systematic review and meta-analysis. Lancet Glob Health Februar. 2017;5(2):e147–56. 10.1016/S2214-109X(17)30006-2 28104184

[3] Yon Y, Ramiro-Gonzalez M, Mikton CR, Huber M, Sethi D. The prevalence of elder abuse in institutional settings: a systematic review and meta-analysis. Eur J Public Health 1 Februar. 2019;29(1):58–67. 10.1093/eurpub/cky093 PMC6359898 29878101

[4] Brijoux T, Neise M, Zank S. Elder abuse in the oldest old: prevalence, risk factors and consequences. Z Gerontol Geriatr. 2021;54(Suppl 2):132–7. 34331085 10.1007/s00391-021-01945-0 PMC8551100

[5] Pillemer K, Burnes D, Riffin C, Lachs MS. Elder Abuse: Global Situation, Risk Factors, and Prevention Strategies. Gerontologist April. 2016;56(Suppl 2):S194–205. 10.1093/geront/gnw004 PMC5291158 26994260

[6] Chadha S, Kamenov K, Cieza A. The world report on hearing, 2021. Bull World Health Organ 1 April. 2021;99(4):242–A242. 10.2471/BLT.21.285643 PMC8085630 33953438

[7] Cosh S, Helmer C, Delcourt C, Robins TG, Tully PJ. Depression in elderly patients with hearing loss: current perspectives. Clin Interv Aging. 2019;14:1471–80. 31616138 10.2147/CIA.S195824 PMC6698612

[8] Deal JA, Reed NS, Kravetz AD, Weinreich H, Yeh C, Lin FR. u. a. Incident Hearing Loss and Comorbidity: A Longitudinal Administrative Claims Study. JAMA Otolaryngol Head Neck Surg 1 Januar. 2019;145(1):36–43. 10.1001/jamaoto.2018.2876 PMC6439817 30419134

[9] Gopinath B, Schneider J, McMahon CM, Teber E, Leeder SR, Mitchell P. Severity of age-related hearing loss is associated with impaired activities of daily living. Age Ageing März. 2012;41(2):195–200. 10.1093/ageing/afr155 22130560

[10] Trott M, Smith L, Xiao T, Veronese N, Koyanagi A, Jacob L. u. a. Hearing impairment and diverse health outcomes: An umbrella review of meta-analyses of observational studies. Wien Klin Wochenschr Oktober. 2021;133(19–20):1028–41. 10.1007/s00508-021-01893-0 34159450

[11] Gaffney HJ, Hamiduzzaman M. Factors that influence older patients’ participation in clinical communication within developed country hospitals and GP clinics: a systematic review of current literature. PLoS One. 2022;17(6):e0269840. 35759474 10.1371/journal.pone.0269840 PMC9236261

[12] Prieur Chaintré A, Couturier Y, Nguyen THT, Levasseur M. Influence of Hearing Loss on Social Participation in Older Adults: Results From a Scoping Review. Res Aging Januar. 2024;46(1):72–90. 10.1177/01640275231174561 PMC10666503 37157996

[13] Courtin E, Knapp M. Social isolation, loneliness and health in old age: a scoping review. Health Soc Care Community Mai. 2017;25(3):799–812. 10.1111/hsc.12311 26712585

[14] Stuth S. Old Age in Germany (D80+): User Manual of SUF D80+, Version 1.0. German Centre of Gerontology; 2023. Verfügbar unter: https://www.dza.de/fileadmin/dza/Dokumente/FDZ/FDZ-D80_Doku/D80_User_Manual_EN.pdf.

[15] Albrecht A, Kaspar R, Simonson J, Stuth S, Hameister N, Tesch-Römer C. Old age in Germany (D80+): representative survey 2020. scientific use file version 1.0. Berlin: Research Data Centre of the German Centre of Gerontology; 2022 [zitiert 9. Juli 2025]. Verfügbar unter: https://www.dza.de/fileadmin/dza/Dokumente/FDZ/FDZ-D80_Doku/D80_Instruments_complete_EN.pdf.

[16] Neise M, Brijoux T, Zank S. Development of the Elder Abuse and Emotional Consequences Scale (EACS). GeroPsych. 2023;36(3):150–60.

[17] Marsden PV. Core discussion networks of Americans. Am Sociol Rev. 1987;52(1):122–31.

[18] Maher RL, Hanlon J, Hajjar ER. Clinical consequences of polypharmacy in elderly. Expert Opin Drug Saf Januar. 2014;13(1):57–65. 10.1517/14740338.2013.827660 PMC3864987 24073682

[19] Katz S, Ford AB, Moskowitz RW, Jackson BA, Jaffe MW. Studies of illness in the aged. The index of ADL: A standardized measure of biological and psychosocial function. JAMA 21 September. 1963;185:914–9. 10.1001/jama.1963.03060120024016 14044222

[20] Lawton MP, Brody EM. Assessment of older people: self-maintaining and instrumental activities of daily living. Gerontologist. 1969;9(3):179–86. 5349366

[21] Kalbe E, Kessler J, Calabrese P, Smith R, Passmore AP, Brand M. u. a. DemTect: a new, sensitive cognitive screening test to support the diagnosis of mild cognitive impairment and early dementia. Int J Geriatr Psychiatry Februar. 2004;19(2):136–43. 10.1002/gps.1042 14758579

[22] Heidenblut S, Zank S. Screening for depression in old age with very short instruments: the DIA-S4 compared to the GDS5 and GDS4. Gerontology Geriatr Med. 2020;6:2333721420981328. 10.1177/2333721420981328 PMC7734509 33354593

[23] Cohen J. Statistical Power Analysis for the Behavioral Sciences. 2. Aufl. New York: Routledge; 1988. 567 S.

[24] Dong X, Chen R, Simon MA. Elder abuse and dementia: a review of the research and health policy. Health Aff Millwood. 2014;33(4):642–9. 24711326 10.1377/hlthaff.2013.1261 PMC9950800

[25] Thennavan S, Selvamani Y, Rangasamy N, Arumai M. Association between Multimorbidity and Psychological Distress among Older Adults in India: The Moderating Role of Elder Abuse. Clin Gerontol. 2025;48(5):1235–45. 10.1080/07317115.2024.2309942 38315752

[26] Podnieks E, Anetzberger GJ, Wilson SJ, Teaster PB, Wangmo T. WorldView Environmental Scan on Elder Abuse. J Elder Abuse Negl Januar. 2010;22(1–2):164–79. 10.1080/08946560903445974 20390830

[27] Yan E, Kwok T. Abuse of older Chinese with dementia by family caregivers: an inquiry into the role of caregiver burden. Int J Geriatr Psychiatry Mai. 2011;26(5):527–35. 10.1002/gps.2561 20690132

[28] von Essen L. Proxy ratings of patient quality of life–factors related to patient-proxy agreement. Acta Oncol. 2004;43(3):229–34. 15244245 10.1080/02841860410029357

[29] Jönsson L, Andreasen N, Kilander L, Soininen H, Waldemar G, Nygaard H. u. a. Patient- and proxy-reported utility in Alzheimer disease using the EuroQoL. Alzheimer Dis Assoc Disord. 2006;20(1):49–55. 16493236 10.1097/01.wad.0000201851.52707.c9

[30] Dinglas VD, Gifford JM, Husain N, Colantuoni E, Needham DM. Quality of life before intensive care using EQ-5D: patient versus proxy responses. Crit Care Med Januar. 2013;41(1):9–14. 10.1097/CCM.0b013e318265f340 PMC3531666 23232287

[31] Coughlin SS. Recall bias in epidemiologic studies. J Clin Epidemiol. 1990;43(1):87–91. 2319285 10.1016/0895-4356(90)90060-3

