# Supplementary material for: Elder abuse and the association with ear or hearing diseases in advanced age: a cross-sectional study
Source: BMC Geriatr. 2026 Feb 24;26:427. doi: 10.1186/s12877-026-07222-2 (PMC13033220; doi:10.1186/s12877-026-07222-2)

Supplement to “Elder abuse and the impact of ear or hearing diseases: a cross-sectional study” by Prell et al.

Supplementary Table 1. Weighted Elder Abuse and Emotional Consequences Scale (EACS) subscales in people with and without ear or hearing diseases (EHD).

**EACS subscales**	**No EHD**		**EHD**	
	**M**	**SD**	**M**	**SD**
Intimidation (items 1 and 2)	2.67_a	1.12	2.87_b	1.19
Shaming and blaming (items 3,4 and 15)	2.44_a	0.88	2.57_b	0.95
Paternalism (items 5 and 6)	2.62_a	1.08	2.78_b	1.14
Neglect (items 7 and 8)	2.41_a	1.00	2.60_b	1.11
Financial exploitation (items 9 and 10)	1.99_a	0.71	2.10_b	0.86
Physical behavior (items 12 and 13)	1.97_a	0.50	2.03_b	0.64

Note: Values in the same row and sub-table where the subscript is not identical, differ at p <.05 in the U-test.

Supplement to "Elder abuse and the impact of ear or hearing diseases: a cross-sectional study" by Prell et al.

Supplementary Table 2. Frequency of Elder Abuse and Emotional Consequences Scale (EACS) patterns in people with and without ear or hearing diseases (EHD).

**EACS subscales**		**No EHD**		**EHD**		
		**N**	**%**	**N**	**%**	**p**
Intimidation	never	671	38.5	312	31.9	<.001
	present	1,071	61.5	665	68.1	
Shaming and blaming	never	995	57.1	492	50.2	<.001
	present	749	42.9	489	49.8	
Paternalism	never	711	41.0	348	35.6	0.006
	present	1,023	59.0	629	64.4	
Neglect	never	945	54.5	455	46.6	<.001
	present	788	45.5	522	53.4	
Financial exploitation	never	1,474	84.7	776	79.2	<.001
	present	266	15.3	204	20.8	
Physical behavior	never	1,560	89.7	854	87.1	0.047
	present	180	10.3	126	12.9	

Note: Group comparison of the presence or absence of elder abuse (rarely, sometimes, frequently, or very often) and no elder abuse (never) in participants with or without ear or hearing diseases.

Supplement to "Elder abuse and the impact of ear or hearing diseases: a cross-sectional study" by Prell et al.

Supplementary Table 3. Elder Abuse and Emotional Consequences Scale (EACS) items in people with and without ear or hearing diseases (EHD).

EACS items		No EHD		EHD	
		N	%	N	%
become louder (item 1)	Never	805	46.3	379	38.8
	Rarely	645	37.1	397	40.7
	Sometimes	240	13.8	155	15.9
	Frequently	43	2.5	42	4.3
	Very often	7	0.4	3	0.3
become abusive (item 2)	Never	1,014	58.2	512	52.3
	Rarely	550	31.6	328	33.5
	Sometimes	145	8.3	109	11.1
	Frequently	27	1.5	27	2.8
	Very often	6	0.3	3	0.3
talked about weaknesses (item 3)	Never	1,286	73.7	666	67.9
	Rarely	345	19.8	255	26.0
	Sometimes	94	5.4	51	5.2
	Frequently	14	0.8	8	0.8
	Very often	5	0.3	1	0.1
blamed for an event (item 4)	Never	1,214	69.6	615	62.7
	Rarely	402	23.1	262	26.7
	Sometimes	108	6.2	83	8.5
	Frequently	19	1.1	18	1.8
	Very often	1	0.1	3	0.3
opinion ignored (item 5)	Never	815	47.0	412	42.2
	Rarely	666	38.4	368	37.7
	Sometimes	215	12.4	168	17.2
	Frequently	33	1.9	23	2.4
	Very often	4	0.2	5	0.5
waive wish or right (item 6)	Never	1,064	61.2	534	54.5
	Rarely	494	28.4	327	33.4
	Sometimes	150	8.6	103	10.5
	Frequently	29	1.7	13	1.3
	Very often	1	0.1	2	0.2
did not give support (item 7)	Never	1,173	67.7	597	61.0
	Rarely	415	23.9	279	28.5
	Sometimes	124	7.2	89	9.1
	Frequently	19	1.1	12	1.2
	Very often	2	0.1	2	0.2
did not give time (item 8)	Never	1,104	63.6	551	56.4
	Rarely	474	27.3	300	30.7
	Sometimes	136	7.8	99	10.1
	Frequently	18	1.0	22	2.3
	Very often	3	0.2	5	0.5
shared use of assets or prop (item 9)	Never	1,581	90.9	845	86.2
	Rarely	112	6.4	98	10.0
	Sometimes	34	2.0	25	2.6
	Frequently	9	0.5	9	0.9
	Very often	4	0.2	3	0.3
to be endured (item 10)	Never	1,536	88.2	828	84.4
	Rarely	159	9.1	107	10.9
	Sometimes	36	2.1	35	3.6
	Frequently	8	0.5	9	0.9
	Very often	3	0.2	2	0.2
touched firmly or roughly (item 11)	Never	1,603	92.1	886	90.5
	Rarely	125	7.2	71	7.3
	Sometimes	11	0.6	19	1.9
	Frequently	0[1]	0.0	2	0.2
	Very often	2	0.1	1	0.1
physically rude (item 12)	Never	1,639	94.1	894	91.2
	Rarely	90	5.2	73	7.4
	Sometimes	9	0.5	9	0.9
	Frequently	2	0.1	4	0.4
	Very often	1	0.1	0[1]	0.0
restricted freedom of movements (item 13)	Never	292	84.6	227	81.1
	Rarely	40	11.6	44	15.7
	Sometimes	10	2.9	8	2.9
	Frequently	3	0.9	1	0.4
medication given without consent (item 14)	Never	337	97.1	271	96.4
	Rarely	9	2.6	9	3.2
	Sometimes	0[1]	0.0	1	0.4
	Frequently	1	0.3	0[1]	0.0
offensive behavior (item 15)	Never	1,525	87.4	833	84.9
	Rarely	184	10.6	131	13.4
	Sometimes	32	1.8	12	1.2
	Frequently	2	0.1	4	0.4
	Very often	1	0.1	1	0.1

Supplement to “Elder abuse and the impact of ear or hearing diseases: a cross-sectional study” by Prell et al.

Supplementary Table 4. Elastic net linear regression, EACS sum score.

Predictor	Coefficient	Standard Error	p
Intercept	33.900	2.960	<.001
Ear or hearing diseases (Yes)	0.903	0.230	<.001
Age	-0.130	0.030	<.001
Gender (Female)	0.174	0.252	0.491
Education: Medium	-0.035	0.365	0.924
Education: High	-0.234	0.395	0.553
Living Alone	1.790	0.623	0.004
Social Network	-0.007	0.015	0.639
Number of medication	0.034	0.034	0.322
Functional health (ADL)	-1.580	0.502	0.002
Functional health (IADL)	-1.110	0.357	0.002
Depression (DIA-S4: 2 – 4 points)	2.120	0.248	<.001
Cognition (semantic fluency)	-0.021	0.018	0.230
Cognition (delayed Recall)	-0.042	0.044	0.335

ADL: basic activities of daily living; DIA-S4: Depression In old Age Scale with 4 items; EACS: Elder Abuse and Emotional Consequences Scale; EHD: ear or hearing diseases; IADL: instrumental activities of daily living.

Supplement to “Elder abuse and the impact of ear or hearing diseases: a cross-sectional study” by Prell et al.

Supplementary Table 5. Multivariate Analysis of Covariances (MANCOVA), parameter estimation.

Independent variable (EACS subscales)	Parameter	Coefficient B	Standard Error	T	p	95% Confidence Inteval		partial η^2
						lower bound	upper bound	
Intimidation	Intercept	2.879	0.037	78.376	<.001	2.807	2.951	0.698
	[EHD = 0]	-0.217	0.046	-4.729	<.001	-0.307	-0.127	0.008
	[EHD = 1]	0[a]						
Shaming and Blaming	Intercept	2.573	0.029	88.354	<.001	2.516	2.630	0.746
	[EHD = 0]	-0.147	0.036	-4.032	<.001	-0.218	-0.075	0.006
	[EHD = 1]	0[a]						
Paternalism	Intercept	2.777	0.035	78.253	<.001	2.707	2.847	0.697
	[EHD = 0]	-0.164	0.044	-3.692	<.001	-0.251	-0.077	0.005
	[EHD = 1]	0[a]						
Neglect	Intercept	2.600	0.033	78.070	<.001	2.535	2.666	0.696
	[EHD = 0]	-0.192	0.042	-4.599	<.001	-0.273	-0.110	0.008
	[EHD = 1]	0[a]						
Financial Exploitation	Intercept	2.100	0.025	85.375	<.001	2.052	2.148	0.733
	[EHD = 0]	-0.114	0.031	-3.714	<.001	-0.175	-0.054	0.005
	[EHD = 1]	0[a]						
Physical Behaviour	Intercept	2.023	0.018	114.414	<.001	1.989	2.058	0.831
	[EHD = 0]	-0.059	0.022	-2.668	0.008	-0.102	-0.016	0.003
	[EHD = 1]	0[a]						

Note: (a): This parameter has been set to zero because it is redundant. EACS: Elder Abuse and Emotional Consequences Scale; EHD: ear or hearing diseases.

Supplement to “Elder abuse and the impact of ear or hearing diseases: a cross-sectional study” by Prell et al.

Supplementary Figure 1. Elder Abuse and Emotional Consequences Scale (EACS) items in people with and without ear or hearing diseases (EHD).

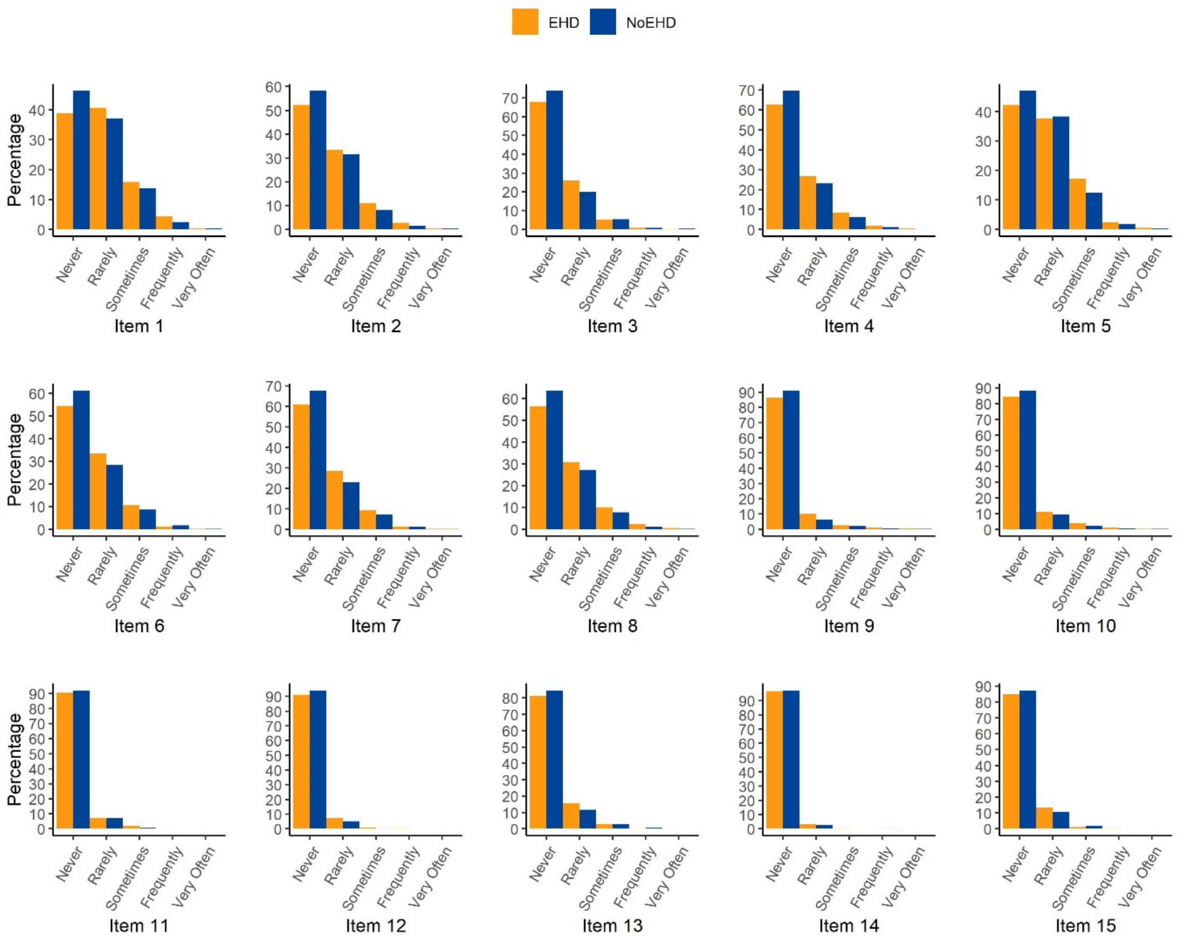

Supplement: Supplementary file 1 — Supplementary Material 1. [file 12877_2026_7222_MOESM1_ESM.pdf]
